# The 5HT4R agonist velusetrag efficacy on neuropathic chronic intestinal pseudo-obstruction in PrP-SCA7-92Q transgenic mice

**DOI:** 10.3389/fphar.2024.1411642

**Published:** 2024-07-30

**Authors:** Yongqiang Liu, Yunfei Wu, Dewan Ren, Yulong Tao, Fangyi Mai, Jingyi Zhu, Xiang Li, Emanuela Colla, Maria Grimaldi, Roberto Giovannini, Fabrizio Giorgi, Loredana Vesci

**Affiliations:** ^1^ Department of Pharmacology, Discovery Services, BioDuro-Sundia, Shanghai, China; ^2^ Department of Human Sciences and Promotion of Quality of Life, San Raffaele Open University, Rome, Italy; ^3^ BIO@SNS, Laboratory of Biology, Scuola Normale Superiore, Pisa, Italy; ^4^ Research and Development, Alfasigma s.p.a., Rome, Italy

**Keywords:** velusetrag, 5-HT4R agonist, transgenic mice, neurodegeneration, rare disease, neuronal intranuclear inclusion disease, ataxin-7

## Abstract

**Background:**

Chronic intestinal pseudo-obstruction (CIPO) is a type of intestinal dysfunction with symptoms of intestinal blockage but without the actual mechanical obstruction. Currently, there are no drugs available to treat this disease. Herein, we report the characterization of the PrP-SCA7-92Q transgenic (Tg) line as a valuable CIPO mouse model and investigated the tolerability and efficacy of the 5-hydroxytryptamine type-4 receptor (5HT4R) agonist velusetrag as a promising pharmacological treatment for CIPO.

**Methods:**

To test the pharmacodynamics of velusetrag, 8-week-old SCA7 Tg mice, which express human mutated *Ataxin-7* gene containing 92 CAG repeats under the mouse prion protein promoter, were treated for 5 weeks by oral route with velusetrag at 1 and 3 mg/kg doses or vehicle. Body weight was monitored throughout the treatment. After sacrifice, the small intestine and proximal colon were collected for whole-mount immunostaining. Untreated, age-matched, C57BL/6J mice were also used as controls in comparison with the other experimental groups.

**Results:**

Analysis of SCA7 Tg mice showed tissue damage and alterations, mucosal abnormalities, and ulcers in the distal small intestine and proximal colon. Morphological changes were associated with significant neuronal loss, as shown by decreased staining of pan-neuronal markers, and with accumulation of ataxin-7-positive inclusions in cholinergic neurons. Administration of velusetrag reversed intestinal abnormalities, by normalizing tissue damage and re-establishing the normal level of glia/neuron’s count in both the small and large intestines.

**Conclusion:**

We demonstrated that the PrP-SCA7-92Q Tg line, a model originally developed to mimic spinocerebellar ataxia, is suitable to study CIPO pathology and can be useful in establishing new therapeutic strategies, such as in the case of velusetrag. Our results suggest that velusetrag is a promising compound to treat patients affected by CIPO or intestinal dysmotility disease.

## 1 Introduction

Neuronal intranuclear inclusion disease (NIID) and polyglutamine (polyQ) disorders are neurodegenerative disorders with shared clinical and pathological features, including the presence of NIIs composed in part of glutamine-rich proteins (Liufu et al., 2022). Similar to polyQ disorders, symptoms of NIID can affect both the CNS and PNS, including the enteric nervous system and can manifest as ataxia, tremor, lower motor neuron impairment, and autonomic deficit (Liu et al., 2002; Tai et al., 2023). Large eosinophilic NIIs can be readily detectable within neuronal nuclei and are associated with a variable extent of neuronal loss. GI abnormalities such as impaired gut motility, dysphagia, and gastroparesis can be severe and can affect the whole tract (Chen et al., 2020). Sporadic episodes of chronic intestinal pseudo-obstruction (CIPO) where peristalsis dysfunction is associated with symptoms of intestinal obstruction without apparent mechanical blockage can be a rare condition in NIIDs but deleterious when present (Goldstein et al., 2016). The onset of CIPO is generally insidious, with gastrointestinal symptoms preceding the first acute episode and symptoms worsening significantly during acute, repeated episodes. Between acute attacks, patients may be asymptomatic or may be affected by persistent gastrointestinal obstruction. Enteric neuropathy, gliopathy, and/or myopathy are all histopathological conditions found in adult and pediatric cases of CIPO (Zenzeri et al., 2020).

Similar to NIIDs, some polyQ disorders such as spinocerebellar ataxia type 7 (SCA7) are characterized by GI abnormalities as well, although CIPO has not been reported in those cases (Velazquez-Perez et al., 2015; Alfonso Ribeiro et al., 2021; Liufu et al., 2022). In SCA7, CAG trinucleotide expansion in the ataxin-7 gene (*ATXN7*) results in the accumulation of a poliQ stretch at the N-terminal of the protein and longer CAG trinucleotide expansions are correlated with early onset and greater overall severity of the disease (Goswami et al., 2022). Ataxin-7 functions as a subunit of the Spt Ada Gcn5 acetyltransferase (SAGA) multiprotein complex, and it is involved in chromatin remodeling upon gene activation and in the differentiation of photoreceptors and Purkinje neurons (Niewiadmska-Cimicka et al., 2019). The protein is ubiquitously expressed, and upon CAG repeat expansion, misfolded ataxin-7 accumulates in intranuclear neuronal inclusions, forming insoluble NIIs. NIIs can be found predominantly in the CNS but also in peripheral locations such as skeletal and cardiac muscle, kidney, and GI tract (Cancel et al., 2000; Trang et al., 2015). Selective overexpression of mutated ataxin-7 in animal models has been associated with selective loss of vulnerable subsets of neurons, associated with progressive ataxia, retinal and cerebellar pathology, and early death (Karam et al., 2018).

Similar to polyQ disorders and SCA7, multiple recent reports identified non-coding CGG repeat expansions in the 5′ region of NOTCH2NLC (Notch 2 N-terminal like C) gene as a cause of NIIDs (Deng et al., 2019; Ishiura et al., 2019; Sone et al., 2019; Tian et al., 2019). Such CGG repeat expansion was found in families affected by adult or juvenile forms of the disease, providing a genetic confirmation for the inheritance and development of NIIDs and further confirming the clinical and pathological resemblance with polyQ diseases.

Until now, the lack of established animal models for NIIDs has limited the progress of effective treatments.

Some years ago, in an effort to model NIIDs and CIPO, Clarke et al. showed that a transgenic (Tg) mouse model for SCA7, the PrP-SCA7-92Q line that expresses human ataxin-7 with an expanded polyQ tract under the prion protein promoter, could be a suitable model for GI neuropathy, as seen in NIID patients (Clarke et al., 2007). Adult SCA7 Tg mice generated by Clarke died prematurely due to intestinal distension and enterocolitis, and this was associated with selective loss of nitric oxide synthase (NOS)-positive neurons, loss of nerve fibers in the myenteric nerve plexus, and delayed GI transit. Accumulation of NIIs composed of ataxin-7 was selectively observed in cholinergic neurons.

In line with these findings and in the attempt to search for a treatment that could improve GI conditions and CIPO in NIIDs, we have investigated, in this work, whether modulation of 5-hydroxytryptamine type-4 receptor (5-HT4R) with velusetrag, a highly selective 5-HT4R agonist, could have a positive effect on intestinal abnormalities seen in young SCA7 mice.

Serotonin (5-HT) is believed to play a pivotal role in the functional regulation of the GI tract (Mawe et al., 2012). 5-HT4 agonists acting on 5-HT4R located on the epithelium, smooth muscle cells, and intrinsic primary afferent neurons can directly or indirectly initiate the peristaltic reflex through the release of acetylcholine (ACh), resulting in decreased colonic transit time, improved bowel movement frequency, and ameliorative bowel satisfaction. Modulation of 5-HT4R with agonists is used to treat GI disorders that require stimulation of the propulsive activity (Hoffman et al., 2012).

Velusetrag (previously known as TD-5108) has shown good potency (pEC50 = 8.3) and high intrinsic activity with human recombinant 5-HT4R and with the native form of 5-HT4 receptors in human and rodent GI tissue (Smith et al., 2008; Abell et al., 2023). Importantly, in contrast to previous drugs, velusetrag did not show any significant affinity for the hERG potassium channel (IC_50_ > 3 mM) or with other 5-HT receptor subtypes (Beattie et al., 2013). Treatment with velusetrag improved GI dysfunction significantly in patients with chronic idiopathic constipation and gastroparesis (Goldberg et al., 2010; Kuo et al., 2021; Abell et al., 2023). In addition, chronic administration of velusetrag in a mouse model of neuronal degeneration induced by overexpression of alpha-synuclein significantly counteracted constipation, intestinal inflammation, dysbiosis, and axonal degeneration in the colon, suggesting that besides being a prokinetic drug, velusetrag could have potentially anti-inflammatory and pro-regenerative properties (Grigoletto et al., 2023). Therefore, because of high tolerability and specificity of velusetrag in treating chronic intestinal constipation, we decided to explore its therapeutic potential for CIPO in a SCA7 Tg mouse model.

## 2 Materials and methods

### 2.1 Mice

The procedures involving the care and use of animals in this study were reviewed and approved by the Institutional Animal Care and Use Committee (IACUC) of BioDuro Shanghai prior to execution.

For model development, the “Mouse prion protein (PRNP) promoter-Kozak-Human ATXN7 CDS-c92Q-Mouse PRNP 3′UTR and 2.2 kb downstream region” cassette was cloned into PV001VT (construct map detailed in Supplementary Figure S1). The plasmid carrying the *ATXN7* transgene was injected into fertilized eggs. The pups were genotyped by PCR to identify those carrying the desired transgene. The following primers were used to assess the presence of human *ATXN7*: Forward 5′-GCA​AGT​GGA​AGC​AAC​CGT​TC-3′ and reverse 5′-TGA​ATG​GGA​TGC​ATT​GGC​CT-3’.

Mice were fed with regular chow (irradiated, Shanghai SLAC Laboratory Animal Co. Ltd., China) plus regular drinking water (municipal tap water filtered using the Mol ultrapure Water System).

At the age of 8 weeks, female/male Tg mice were treated with either a vehicle or velusetrag at 1 mg/kg or 3 mg/kg in the dissolved saline solution for 5 weeks. The normal, age-matched mice (C57Bl/6 from Cyagen, China) were not treated with a vehicle or drug and were used as controls.

### 2.2 Gut dilatation measurements

Mice were weighed every 2 days. When euthanizing the animals, the intestinal apparatus was placed on a paper for calibration and photographed. Diameter of five regions for the distal small intestine (DSI) and colon were measured by the “straight line” tool of ImageJ.

### 2.3 Histopathological analysis of DSI and the proximal colon

Tissues were fixed in 4% paraformaldehyde for 48 h, then embedded in paraffin (Wuhan Junjie Electronics Co., Ltd., Wuhan, China), and cut into 4-mm sections (Leica Instrument Shanghai Ltd., Shanghai, China). These slices were dewaxed and then stained with hematoxylin–eosin (HE; ServiceBio Co., Ltd, Wuhan, China), and then photographed and observed under a light microscope (Nikon Eclipse CI-L, Tokyo, Japan) with a ×20 objective. The inflammation score ranged from 0 to 3+ and was assigned as follows: 0, no change; 1+, mild; 2+, moderate; and 3+, severe. The ulcer score ranged from 0 to 3+ and was assigned as follows: 0, no change; 1+, 0%–5% mucosal damage; 2+, 5%–33% mucosal damage; and 3+, above 33% mucosal damage. The bleeding score ranged from 0 to 2+ and was assigned as follows: 0, no change; 1+, 0%–30% area; and 2+, above 30% area. The total score is the sum of above three lesions.

### 2.4 Whole-mount immunostaining

Segments of the distal ileum and proximal colon were placed in phosphate-buffered saline (PBS), and the mucosa and submucosa were manually removed with fine forceps. The muscularis propria and enclosed myenteric plexus were fixed for 10 min in ice-cold acetone and then washed with 1× PBS. Blocking was performed in 1× PBST (0.1% Tween 20) with 1% BSA for 1–3 h at room temperature with gentle rocking before antibody incubation.

The following primary antibodies were used: Hu C/D 1:500 (Abcam, Ab184267, US), Ataxin-7 1:2000 (Thermo Fisher, PA1-749, US), MAP2 1:1,000 (Abcam, ab183830, US), nNOS 1:500 (Abcam, ab76067, US), ChAT 1:1,000 (Abcam, Ab181023, US), Calretinin 1:500 (Merck, MAB1568, US), and SOX10 1:250 (Invitrogen, MA5-32398, US).

Secondary antibodies included F(ab’)2-goat anti-rabbit HRP 1:1,000 (Abcam, Ab 6013, US), donkey anti-Mouse IgG (H + L) highly cross-adsorbed secondary antibody Alexa Fluor 488 1:500 (Invitrogen, A-21202, US), and donkey anti-Rabbit IgG (H + L) highly cross-adsorbed secondary antibody Alexa Fluor 594 1:500 (Invitrogen, A-21207, US). TSA520 1:200 (Wi See Biotechnology, D11013, China) and TSA570 1:200 (Wi See Biotechnology, D11011, China) were used for fluorescent signal amplification.

All sections were counterstained with DAPI.

Images were obtained from each animal using a confocal model of the Operetta CLS High Content Analysis System (PerkinElmer, HH16000001, US) with 40× magnification and running Harmony software. For analysis of immunofluorescence staining, cell bodies were counted using the ImageJ automatic tool Cell Counter plugin.

### 2.5 qPCR

To evaluate the expression of 5-HT4R, total RNA was isolated using the TRIzol reagent (Invitrogen, United States) from homogenized small intestine and colon tissues, following the respective manufacturer’s protocol. In addition, 1 μg of total RNA was used for cDNA synthesis with the PrimeScript RT Reagent Kit with gDNA Eraser (Perfect Real Time) (TaKaRa, Japan). The obtained cDNA was used for reverse transcription quantitative PCR (qPCR). RT-qPCR reactions were performed with SYBR Green PCR Master Mix (Thermo Fisher Scientific, Applied Biosystems). The primers were purchased from Shanghai Sangon (China) and are listed in the following table.

**Table:** 

Primer	Sequence (5′-3′)
5HT4R F	ACC​AAG​GCA​GCC​AAG​ACT​TT
5HT4R R	TAG​CCA​AGC​CAG​AGG​AAA​GC
GAPDH F	GAC​ATG​CCG​CCT​GGA​GAA​AC
GAPDH R	AGC​CCA​GGA​TGC​CCT​TTA​GT

Cycle threshold (Ct) values were collected and exported from the Bio-Rad CFX384 Instrument. Relative expression of the 5HT4R gene in each sample was normalized to the housekeeping gene (*GAPDH*) and was calculated using the formula: 2^−ΔCT^ = 2^-(Ct^ 5HT4R–^Ct^ GAPDH^)^. The relative expression level of the 5HT4R gene in vehicle and treated groups to the normal, C57Bl/6 group was calculated using the following formula: 2^−ΔΔCT^ = 2^− (ΔCt^ treated^−ΔCt^ normal).

### 2.6 Statistical analyses

All data were presented as mean ± SEM. Experimental data were analyzed using Graph Pad Prism 9 software. A one-way non-parametric ANOVA, Kruskal–Wallis test, followed by Dunn’s multiple comparisons, was used. *p* < 0.05 or below was considered statistically significant.

## 3 Results

### 3.1 Velusetrag rescues intestinal abnormalities in SCA7 mice

**FIGURE 1:**
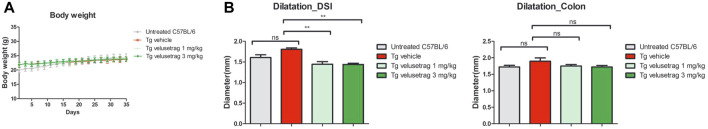
Body weight and intestine dilation assessment in SCA7 mice and C57Bl6 controls after velusetrag treatment. SCA7 Tg mice were treated for 5 weeks with 1 mg and 3 mg/kg of velusetrag or a vehicle. **(A)**. Body weight of Tg and controls; untreated C57Bl6 mice was recorded every 2 days throughout the 5 weeks of treatment. No detectable differences among all four groups were evident. **(B)**. The diameter of the distal small intestine (DSI) and colon of animals at age 13 weeks, after the completion of the pharmacological treatment, was recorded. After sacrifice, each intestine was placed on a paper for calibration, and the diameter of five regions for DSI and the colon was measured. Values in graphs in A and B are presented as the mean ± SEM, n = 10/group, one-way non-parametric ANOVA, Kruskal–Wallis test, followed by Dunn’s multiple comparisons, *p* < 0.01.

To validate velusetrag as possible treatment for CIPO, we initially assessed changes in body weight and gross intestinal morphology in young SCA7 Tg animals treated either with a vehicle or with the 1 or 3 mg/kg dosage throughout the treatment. Untreated, age-matched C57Bl/6 mice were used as controls for the transgene expression. No significant differences were noticed among all groups (Figure 1A). After 5 weeks of treatment, the body weight of SCA7 Tg mice or untreated C57Bl/6 did not show notable differences, showing that velusetrag appeared to be well-tolerated at both dosages. Similarly, when intestinal morphology was examined and compared to untreated C57Bl/6 mice, the dilatation of the distal small intestine (DSI) was found unchanged in Tg mice treated with only a vehicle (Figure 1B) for both DSI and colonic tissues. On the contrary, velusetrag at both doses was able to significantly reduce gut dilation compared to the Tg-vehicle-treated group (1.81 ± 0.01 for the SCA7 Tg-vehicle group vs. 1.44 ± 0.02 for 1 mg and 1.44 ± 0.01 for 3 mg, p < 0.01) but only in DSI.

**FIGURE 2:**
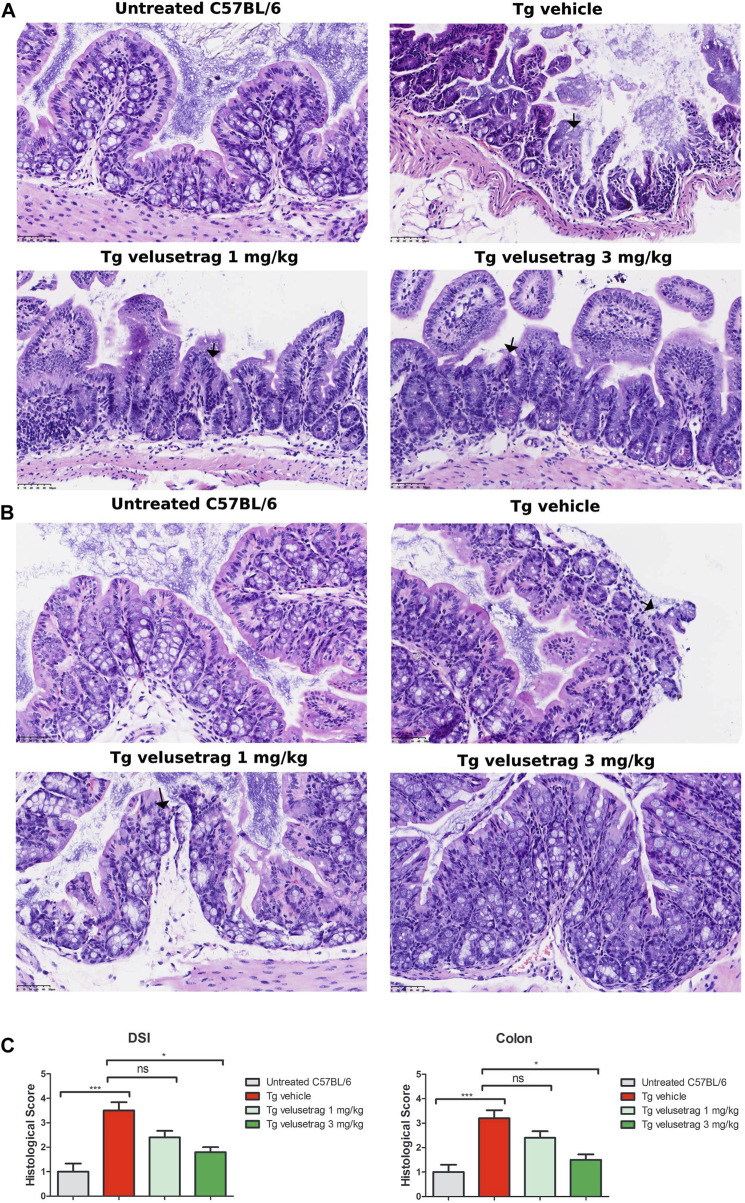
Velusetrag treatment improves intestinal pathology in SCA7 mice. H&E staining of DSI **(A)** and colon **(B)** obtained from 13-week-old untreated C57Bl6 controls and SCA7 Tg mice treated with velusetrag or a vehicle shows that the 5-HT4R agonist improves intestinal abnormalities in both regions at the highest dosage. Mouse tissues were fixed in 4% paraformaldehyde for 48 h and then stained with H&E. Images were acquired with a light microscope with 20× objective. Black arrows point to mucosal damage. **(C)**. Graphs of A and B include histological total score values represented as the mean ± SEM, n = 10, one-way non-parametric ANOVA, Kruskal–Wallis test, followed by Dunn’s multiple comparisons, *p* < 0.05 and *p* < 0.001.

To investigate whether overexpression of ataxin-7 was associated with morphological enteric abnormalities, we performed H&E staining of both DSI and the proximal colon in all groups after 5 weeks of velusetrag treatment (Figures 2A–C). In the untreated C57Bl/6 mice, the mucosa structure was intact, and the villi arranged closely and neatly. In the SCA7 Tg-vehicle-treated group, the intestinal mucosa was severely damaged, and the villi were arranged sparsely and discontinued (in DSI, 3.5 ± 0.1 for Tg-vehicle vs. 1.0 ± 0.1 for untreated C57BL/6, p < 0.001; in colon, 3.2 ± 0.1 for Tg-vehicle vs. 1.0 ± 0.1 for untreated C57BL/6, p < 0.001). Velusetrag administration, especially at the highest dosage, attenuated the damage to the small intestinal mucosa and to the colon (in DSI, it was 2.4 ± 0.1 for 1 mg and 1.8 ± 0.1 for 3 mg, p < 0.05; in colon, it was reduced to 2.4 ± 0.1 for 1 mg and 1.5 ± 0.1 for 3 mg, p < 0.05). These findings showed that the SCA7 Tg line is characterized by severe intestinal damage with the presence of ulcers and mucosal lesions, whereas administration of velusetrag effectively improves intestinal tissue morphology.

### 3.2 Velusetrag treatment improves neuronal loss in the intestine of SCA7 mice

**FIGURE 3:**
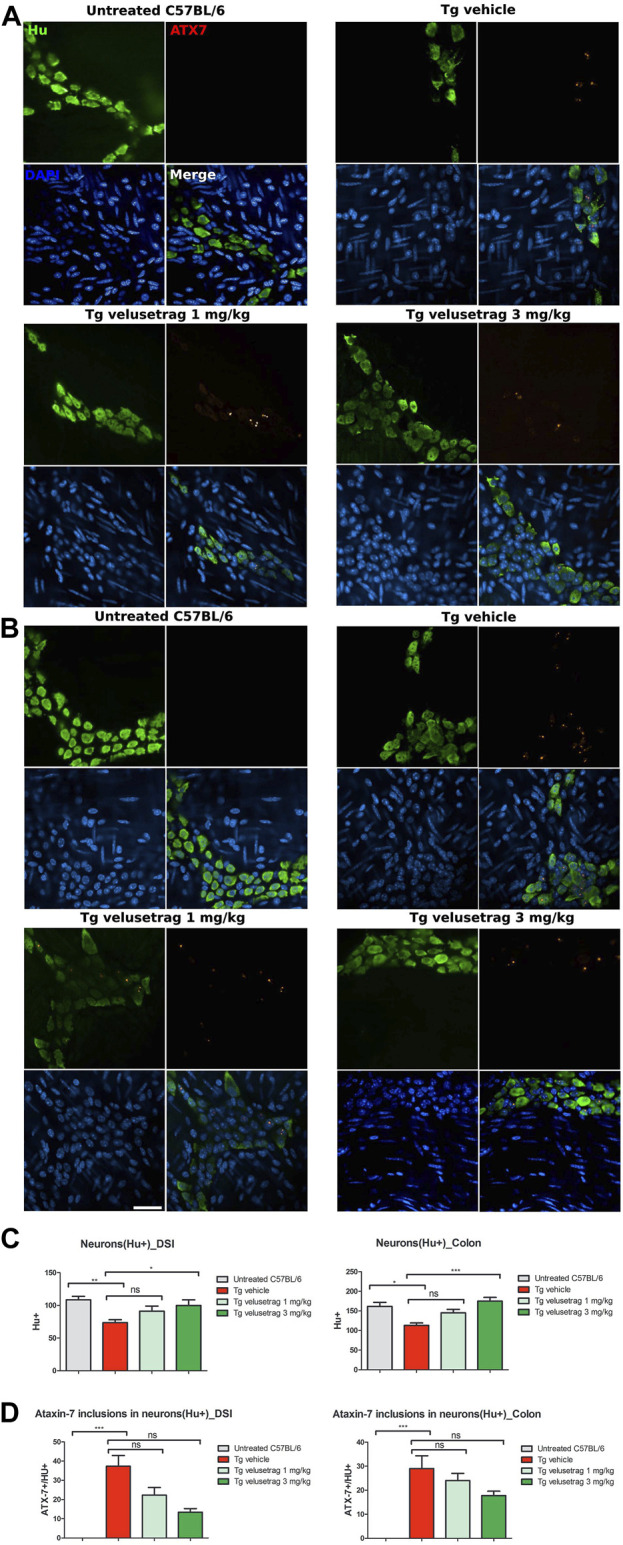
Velusetrag treatment improves neuronal loss in the intestine of SCA7 mice. Immunofluorescence staining with a neuronal specific marker, Hu C/D, and ataxin-7 shows that velusetrag treatment ameliorates neuronal loss when given at the highest dosage in both DSI **(A)** and the colon **(B)**. Whole-mount sections of DSI and the colon were prepared by removing the mucosal and submucosal layers. Tissues were fixed in acetone and stained with Hu C/D and ataxin-7 (ATX-7). Co-localization of ataxin-7 as intranuclear inclusions with Hu C/D+ cytoplasmic markers was observed in SCA7 Tg mice in both DSI and the colon. Images were acquired using a confocal model of the Operetta CLS High Content Analysis System with 40× objective. For analysis of immunofluorescence staining, cell bodies were counted using the ImageJ automatic tool Cell Counter plugin. Scale bar = 50 μm. **(C)**. Quantitative analysis of Hu C/D^+^ neurons in DSI and the colon. Values on the graphs are represented as the mean ± SEM, n = 10, one-way non-parametric ANOVA, Kruskal–Wallis test, followed by Dunn’s multiple comparisons, p < 0.05, p < 0.01, and p < 0.001; ns, not significant. **(D)** Percentage of co-localization of ATX-7 with Hu C/D^+^ neurons was determined as a ratio between the number of total Hu^+^ neurons and Hu^+^ neurons with ATX-7 inclusions. Values on the graphs are represented as the mean ± SEM, n = 10, one-way non-parametric ANOVA, Kruskal–Wallis test, followed by Dunn’s multiple comparison, p < 0.001; ns, not significant.

To investigate whether intestinal dysfunction in SCA7 Tg mice was associated with neuronal cell loss and accumulation of proteinaceous inclusions, positive for ataxin-7, immunofluorescence was performed on whole-mount preparations of myenteric plexus from DSI and the proximal colon from 13-week-old Tg male mice (Figures 3A–D). Staining with Hu C/D or Map2 (Supplementary Figure S2), two pan-neuronal markers, revealed extensive neuronal loss in both DSI (A) and the colon (B) of vehicle-treated Tg mice when compared to untreated controls (in DSI 108.40. ± 5.41 for C57BL/6 vs. 73.50 ± 4.60 for Tg-vehicle, p < 0.01; in colon 158.9. ± 12.16 for C57BL/6 vs. 113.30. ± 5.92 for Tg-vehicle, p < 0.05). At the same time, double staining with ataxin-7 and Hu C/D revealed that approximately 40% of enteric neurons in DSI and 30% in the proximal colon co-localized with ataxin-7-positive nuclear inclusions.

After 5 weeks of velusetrag administration, the level of neuronal loss was significantly reduced, especially in the colon and with the higher dosage (in DSI, 91.00 ± 1.88 for 1 mg and 99.90 ± 2.68 for 3 mg, p < 0.05; in colon, 145.10 ± 8.93 for 1 mg and 175.20 ± 17.87 for 3 mg, p < 0.001). Concomitantly, the amount of double positive neurons with ataxin-7 inclusions seemed reduced, although not significantly by the administration of the 5-HT4R agonist in a dose-dependent manner (in DSI, 29.08. ± 0.05 for Tg-vehicle vs. 24.13 ± 0.03 for 1 mg velusetrag and 17.87 ± 0.02 for 3 mg/kg).

Desensitization of the 5HT4R agonist was not apparent after 5 weeks of velusetrag treatment, as shown by unchanged mRNA expression levels in DSI and colonic tissues of treated groups (Supplementary Figure S3). Interestingly, vehicle-treated SCA7 Tg mice showed significantly different 5HT4R levels compared with the control C57Bl/6 animals in colonic tissue, a result that could be related to gross structural changes highlighted above for this Tg line and it would need further evaluation.

**FIGURE 4:**
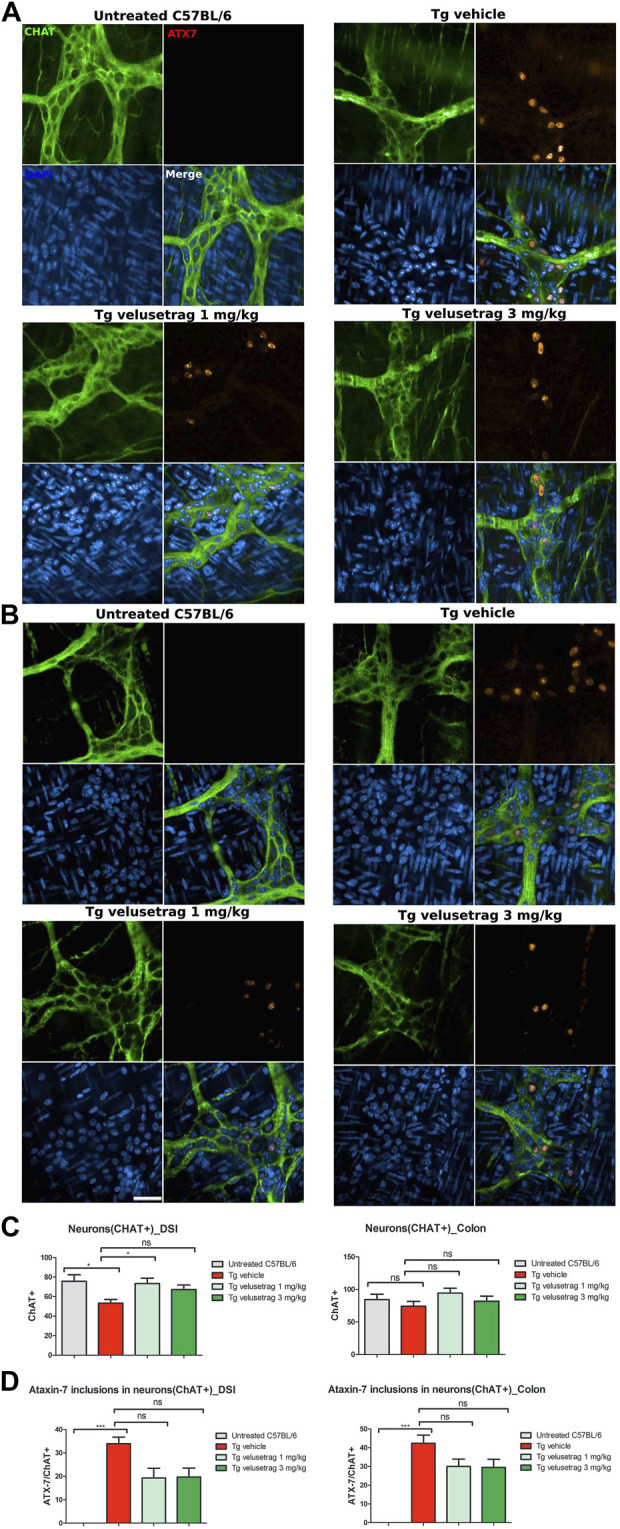
Velusetrag treatment improves cholinergic neuronal survival in the small intestine of SCA7 mice. Immunofluorescence assay with a cholinergic specific marker, CHAT, and ataxin-7 shows that 1-mg velusetrag treatment ameliorates CHAT^+^ neuronal survival in DSI **(A)**. No differences in neuronal count were observed in the colon of SCA7 Tg mice and untreated C57Bl6 **(B)**. Whole-mount sections of DSI and the colon were stained with CHAT and ataxin-7 (ATX-7), as described earlier. Co-localization of ataxin-7 as intranuclear inclusions with CHAT was observed in SCA7 Tg mice in both DSI and the colon. Images were acquired using a confocal model of the Operetta CLS High Content Analysis System with 40× objective. For analysis of immunofluorescence staining, cell bodies were counted using the ImageJ automatic tool Cell Counter plugin. Scale bar = 50 μm. **(C)**. Quantitative analysis of CHAT^+^ neurons in DSI and the colon. Values on the graphs are represented as the mean ± SEM, n = 10, one-way non-parametric ANOVA, Kruskal–Wallis test, followed by Dunn’s multiple comparisons, p < 0.05. **(D)** Percentage of co-localization of ATX-7 with Hu C/D neurons was determined as the ratio between the number of total Hu^+^ neurons and Hu^+^ neurons with ATX-7 inclusions. Values on the graphs are represented as the mean ± SEM, n = 10, one-way non-parametric ANOVA, Kruskal–Wallis test, followed by Dunn’s multiple comparisons, p < 0.001; ns, not significant.

To study more in detail whether neurodegeneration was linked to a specific neuronal population, whole-mount sections of DSI and the colon from all groups were stained with cholinergic (CHAT) and nitrergic (NOS) markers. Interestingly, CHAT^+^ neurons appeared reduced in 13-week-old Tg mice compared to untreated C57Bl/6 only in DSI but not in the colon (Figures 4A,B respectively) and velusetrag treatment resulted in CHAT^+^ neuron recovery already at the lowest dosage. CHAT^+^ neurons were also seen to accumulate ataxin-7 inclusions at comparable percentages in DSI and in the proximal colon in the vehicle-treated group compared to untreated C57Bl/6 mice. With the administration of velusetrag, there was a trend toward a reduction of ataxin-7^+^ inclusions in both DSI and the colon, but it was not significant (in DSI, 0.00. ± 0.00 for untreated C57BL/6 vs. 33.99 ± 0.91 for Tg-vehicle, p < 0.001 19.27 ± 1.30 for 1 mg/kg velusetrag and 19.63 ± 1.20 for 3 mg/kg velusetrag; in colon, 0.00. ± 0.00 for untreated C57Bl6 vs. 42.34 ± 1.37 for Tg-vehicle, 29.93 ± 1.26 for 1 mg/kg velusetrag and 29.47 ± 1.39 for 3 mg/kg velusetrag) (Figures 4A–D).

**FIGURE 5:**
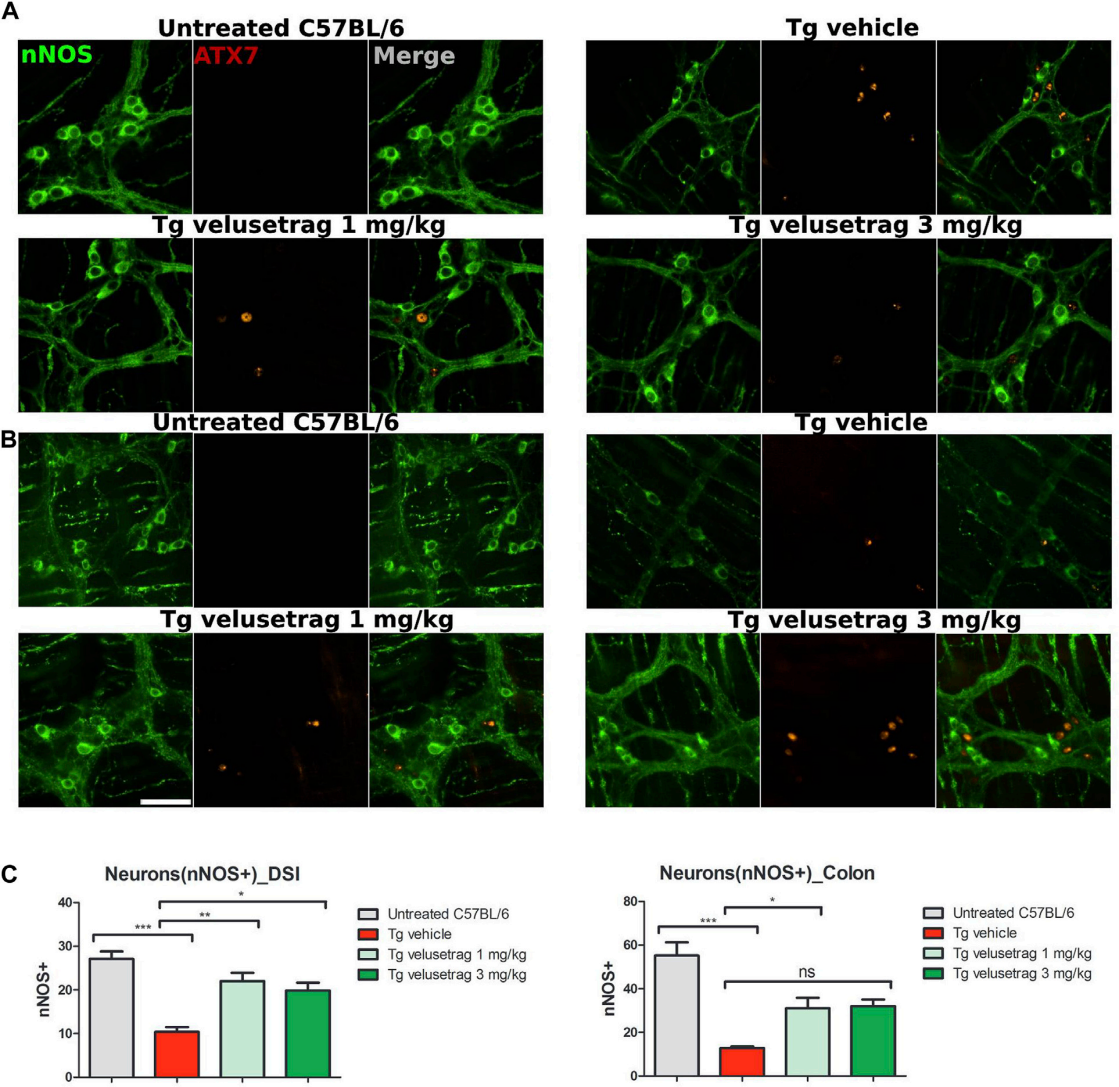
Velusetrag treatment improves nitrergic neuronal survival in the small intestine of SCA7 mice. Immunofluorescence staining of ataxin-7 and nNOS as the specific marker of nitrergic neurons performed in the distal small intestine and colon shows that 1-mg velusetrag treatment improves nNOS^+^ neuronal survival in DSI **(A)** and in the colon **(B)**. Whole-mount sections of DSI and the colon were stained with nNOS and ataxin-7 (ATX-7), as described above. Ataxin-7 immunostaining did not co-localize with nNOS^+^ neurons in SCA7 Tg mice in both DSI and the colon. Images were acquired using a confocal model of the Operetta CLS High Content Analysis System with 40× objective. For analysis of immunofluorescence staining, cell bodies were counted using the ImageJ automatic tool Cell Counter plugin. Scale bar = 50 μm. **(C)**. Quantitative analysis of nNOS^+^ neurons in DSI and the colon. The results are presented as the mean ± SEM, n = 10, one-way non-parametric ANOVA, Kruskal–Wallis test, followed by Dunn’s multiple comparisons. p < 0.05, p < 0.01, and p < 0.001.

Similarly, for the nitrergic system, intestinal pathology in SCA7 mice was associated with a decline in NOS^+^ neurons in both DSI (10.40 ± 0.35 for Tg-vehicle vs. 27.10 ± 0.54 for untreated C57Bl6, p < 0.0001) and colonic tissues (12.80 ± 0.24 for Tg-vehicle vs. 55.20 ± 1.91 for C57Bl6) (Figures 5A–C). No ATX-7^+^ inclusions were detected in these neurons, a characteristic already shown in other SCA7 models (Clarke et al., 2007). Velusetrag treatment significantly re-established a normal level of nitrergic neurons in both DSI (22.00 ± 0.61 for 1 mg/kg velusetrag and 19.90 ± 0.55 for 3 mg/kg, p < 0.01 and p < 0.05, respectively) and colonic tissue (31.00 ± 1.52 for 1 mg/kg velusetrag and 32.00 ± 1.02 for 3 mg/kg dosage, p < 0.05).

**FIGURE 6:**
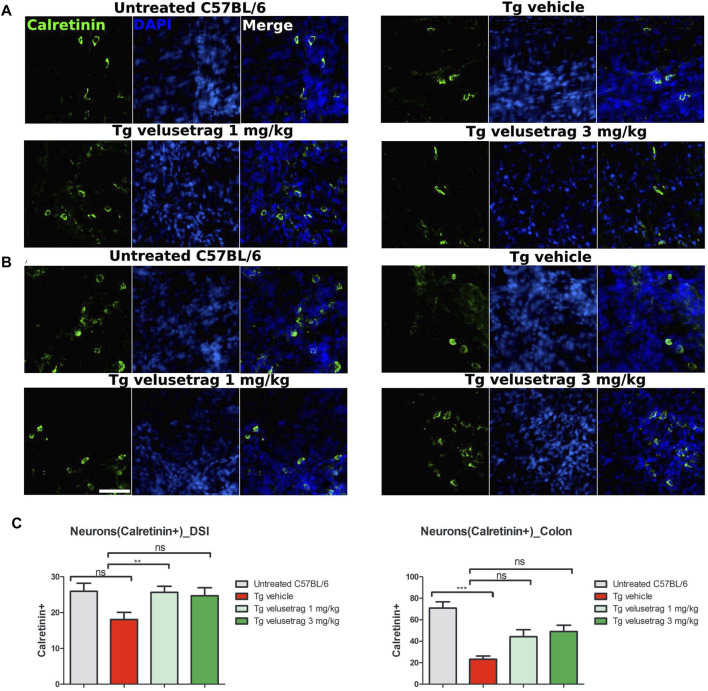
Velusetrag treatment improves calretinin-positive neuronal count in the colon of SCA7 mice. Immunofluorescence staining with calretinin, a marker associated with cholinergic neurons but not with the nitrergic system in the ENS, showed a swift decline of cholinergic neurons in the colon of SCA7 Tg mice **(B)** that was improved by velusetrag at both dosages. No differences in the neuronal count were observed in DSI of SCA7 mice and untreated C57Bl6 **(A)**. Whole-mount sections of DSI and the colon of Tg mice and untreated controls were stained with calretinin. Images were acquired using a confocal model of the Operetta CLS High Content Analysis System with 40× objective. For analysis of immunofluorescence staining, cell bodies were counted using the ImageJ automatic tool Cell Counter plugin. Scale bar = 50 μm **(C)**. Quantitative analysis of calretinin-positive neurons in DSI and the colon. Values on the graphs are represented as the mean ± SEM, n = 10, one-way non-parametric ANOVA, Kruskal–Wallis test, followed by Dunn’s multiple comparison, p < 0.001; ns, not significant.

Decline of the cholinergic system was also confirmed in the colon of SCA7 mice when tissues were immunostained with calretinin, a marker that is associated in the ENS with cholinergic but not nitrergic neurons (Masliukov et al., 2017). Interestingly, vehicle-treated SCA7 Tg mice showed a significant loss of calretinin^+^ neurons in the colon (23.40 ± 0.92 for Tg-vehicle vs. 70.90 ± 1.83 for untreated C57Bl6, p < 0.0001) whereas the decline in DSI was not that apparent (Figures 6A–C). Velusetrag administration seemed to improve the normal calretinin^+^ neurons level, although not significantly, compared to vehicle-treated Tgs (44.30 ± 2.02 for 1 mg and 49.10 ± 1.85 for 3 mg). Taken together, these results show a widespread neuronal degeneration in the intestinal wall of adult SCA7 mice that is specifically counteracted by velusetrag treatment.

### 3.3 Velusetrag treatment re-establishes normal glia/neuron ratio in SCA7 mice

**FIGURE 7:**
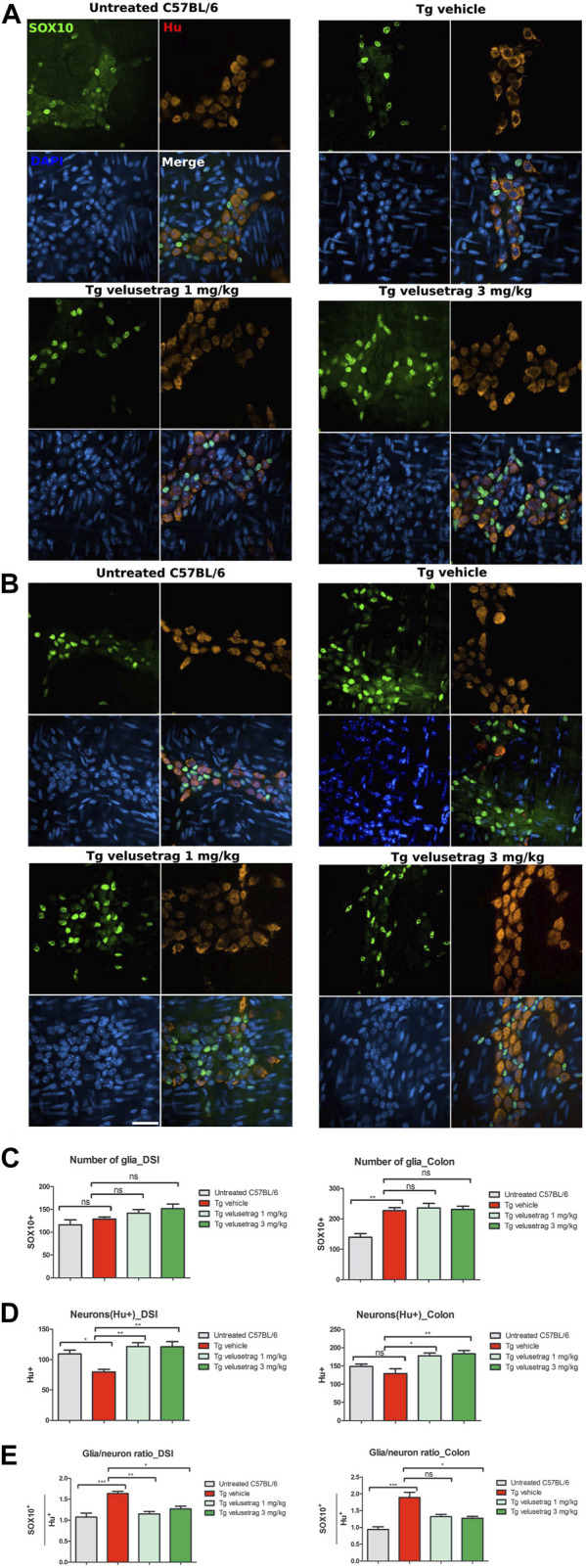
Velusetrag treatment re-establishes normal glia/neuron ratio in the ENS of SCA7 mice. Double staining with SOX10, a marker for glial cells and glial precursors, and Hu C/D showed that velusetrag treatment was able to re-establish normal ratio glia/neurons in the ENS of SCA7 mice. **(A, B)** Whole-mount sections of DSI **(A)** and colon **(B)** of Tg mice and untreated controls were co-stained with SOX10 and Hu C/D and H and imaged with the confocal model of the Operetta CLS High Content Analysis System, with 40× objective Scale bar = 50 μm. **(C, D)** Cell count of SOX10+ and Hu C/D+ cells in DSI and the colon. Values on the graphs are represented as the mean ± SEM, n = 10, one-way non-parametric ANOVA, Kruskal–Wallis test, followed by Dunn’s multiple comparisons, p < 0.05 and p < 0.01. **(E)** Quantitative analysis of glia/neuron ratio in DSI and the colon. Values on the graphs are represented as the mean ± SEM, n = 10, one-way non-parametric ANOVA, Kruskal–Wallis test, followed by Dunn’s multiple comparison; p < 0.05, p < 0.01, and p < 0.001.

Lastly, the intestinal glia population was investigated by counting enteric glia cells stained with SOX10, a marker for glial precursors and fully differentiated glial cells (Britsch et al., 2001) in whole-mount preparations of the myenteric plexus in treated and untreated mice (Figures 7A–E). Vehicle-treated SCA7 Tg mice showed a significant increase in the glia content in the proximal colon (873.85 ± 12.05 for Tg-vehicle vs. 535.77 ± 14.43 for untreated C57Bl6, p < 0.01) but not in DSI. Compared to Hu C/D staining within the same sections, the ratio of SOX10/Hu^+^ neurons was positively increased in both intestinal regions in SCA7 Tg mice compared to C57Bl/6 controls. Although velusetrag has renowned anti-inflammatory properties (Grigoletto et al., 2023), administration of both dosages did not affect the level of SOX10^+^ cells *per se*’ (in colon 905.38 ± 18.96 for 1 mg velusetrag and 888.85 ± 12.46 for 3 mg) but appeared to re-establish normal SOX10/Hu^+^ cell levels as comparable to untreated controls, probably by acting on improving neuronal survival.

## 4 Discussion

CIPO is an intestinal chronic condition, seen in subjects affected by NIIDs, that is difficult to model, and the absence of appropriate systems has limited the development of effective treatments. Very recently, few groups reported the presence of repeated poliQ expansions in the NOTCH2NLC gene as a causative mutation of NIIDs (Deng et al., 2019; Ishiura et al., 2019; Sone et al., 2019; Tian et al., 2019). Such evidence provided a genetic background for NIIDs and reinforced the association of NIIDs with poliQ disorders.

In this work, we have confirmed that SCA7 Tg mice, a model of spinocerebellar ataxia, a poliQ disease, could be a suitable model to investigate CIPO. Since young age, SCA7 Tgs accumulate structural and morphological intestinal abnormalities such as scarring, tissue damage, and mucosal bleeding, which are all associated with extensive loss of enteric neurons in DSI and the proximal colon, especially for neurons belonging to the cholinergic and nitrergic network. In addition, enteric NIIs positive for ataxin-7 were also found in cholinergic neurons.

In the attempt to mitigate intestinal damage in this line, we tested the efficacy of the 5-HT4R agonist velusetrag, a highly selective compound developed to treat chronic constipation and found to be effective in humans and rodents (Golberg et al., 2010; Long et al., 2012; Kuo et al., 2021; Abell et al., 2023). Histopathological analysis of tissues through H&E staining showed that 5 weeks treatment with velusetrag at 1 mg and 3 mg/kg was able to significantly improve the intestinal damage in DSI and colon in a dose-dependent manner. Moreover, velusetrag treatment reduced neuronal loss, as shown by double immunofluorescence with Hu C/D, having a positive effect of both cholinergic (CHAT^+^ and calreticulin^+^) and nitrergic (nNOS^+^) neurons. Although velusetrag’s rescue effect on the cholinergic system was somehow expected as described in other models (Grigoletto et al., 2023), more surprising was the result on the nitrergic system. Both systems represent the major signaling pathways in the ENS, where the cholinergic circuitry mediates motility and secretory reflexes, whereas the nitrergic system is involved in GI smooth muscle relaxation and the regulation of normal peristalsis (Spencer and Hu, 2020). Therefore, alterations in any of them or both, as in the SCA7 mice, result in intestinal motility disarrangement and constipation. Velusetrag treatment had a very extensive effect, acting on both ENS communication systems, suggesting a broad-range neuroprotective properties together with the prokinetic activity. Another supporting evidence of this exhaustive action of velusetrag was the improvement seen in the glia/neuron’s ratio in both intestinal regions after treatment. Notably, such an action seemed specific for the neuronal lineage rather than glia as the number of glia cells in the small and large intestines in SCA7 mice was not affected by administration of the 5HT4R agonist.

Taken together, these results confirm that velusetrag has a pleiotropic effect on ENS homeostasis that can make this drug applicable to many conditions with altered intestinal function and enteric neuropathy.

By considering the histopathology, on the whole, CIPO can be seen as an enteric neuropathy associated with neuronal and muscle degeneration and enteric inflammation (Goldstein et al., 2016). Velusetrag could possibly target different aspects of CIPO pathophysiology, from the abnormal tissue morphology including improving neuronal loss and lowering inflammation to the enteric function involving the stimulation of GI motility and promoting the intestinal wellbeing with a balanced microbial population. Notably, velusetrag has been shown to ameliorate dysbiosis in a mouse model of Parkinson’s disease (Grigoletto et al., 2023). Since an altered gut microbiota represents a risk factor for CIPO (Radocchia et al., 2021), it is possible that this aspect could also be improved by velusetrag administration.

In addition, velusetrag treatment was well-tolerated in rodents despite the chronic administration as shown before (Long et al., 2012; Grigoletto et al., 2023) and 5-HT4R desensitization was not apparent in SCA7 mice for both dosages, as shown by the unchanged receptor mRNA level compared with the vehicle-treated group. Interestingly, the 5-HT4R mRNA level was altered instead when SCA7 mice were compared with untreated C57Bl/6 animals, confirming intestinal alterations of ENS circuitries.

Accumulation of ataxin-7^+^ NIIs was seen in enteric neurons, especially in the cholinergic system (Clarke et al., 2007), a finding that was also confirmed in our SCA7 line. Although this result should be expanded considering other neuronal types besides the cholinergic and nitrergic circuitry in the ENS, intestinal cholinergic neurons could be particularly sensitive to the accumulation of poliQ/ataxin-7 aggregates. Vulnerability of certain neuronal types has already been hypothesized in SCA7 pathology but largely highlighted in the brain and in the retina (Karam et al., 2018).

Although velusetrag administration shows promising and beneficial effects in CIPO, we must consider the overall limitations to this study. SCA7 mice can only partially model CIPO; therefore, the results obtained in this study should be taken cautiously and confirmed possibly in other systems. The lack of genetic or pharmacological models of CIPO has limited research related to pathophysiology and new treatments. New recent genetic data on non-coding CGG repeat expansions in the 5′ region of the NOTCH2NLC gene (Deng et al., 2019; Ishiura et al., 2019; Sone et al., 2019; Tian et al., 2019) confirmed that NIIDs and CIPO can have a genetic origin similar to poli(Q) diseases, and therefore, the SCA7 model cannot be completely ruled out. At the same time, the finding of new genetic targets can pave the way for developing more suitable models of CIPO.

## 5 Conclusion

In this study we have evaluated the effect of the 5HT4R agonist velusetrag to treat intestinal dysfunction in a preclinical model of CIPO, the SCA7 Tg mice. Overall, extended treatment with velusetrag significantly improved intestinal abnormalities, tissue damage, and neuronal loss, therefore holding high therapeutic potentials for treatment of CIPO and other related gastrointestinal disorders in human subjects.

## Data Availability

The original contributions presented in the study are included in the article/Supplementary Material, further inquiries can be directed to the corresponding authors.

## Supplementary material

SUPPLEMENTARY FIGURE S1 Plasmid map of PRP ATXN7 CDS-c92Q construct. The “Mouse prion protein (PRNP) promoter-Kozak-Human ATXN7 CDS-c92Q-Mouse PRNP 3′UTR and 2.2 kb downstream region” cassette was cloned into PV001VT between XmaI and EcoRV sites. Positive clones were screened and expanded.

SUPPLEMENTARY FIGURE S2 Velusetrag treatment improves neuronal loss in the intestine of SCA7 mice. Tissue staining for the pan-neuronal marker MAP2 in DSI **(A)** and the colon **(B)** of SCA7 Tg mice and untreated C57Bl6 controls. Whole-mount sections were stained with MAP2 and counterstained with DAPI. Immunofluorescence assay confirmed significant neuronal decline in DSI, as shown by Hu C/D staining. Images were acquired with a confocal model of Operetta CLS High-Content Analysis System with 40× objective. Scale bar = 50 μm. C. Graphs of quantitative analysis of MAP2 staining. The results are presented as the mean ± SEM, n = 10, one-way non-parametric ANOVA, Kruskal–Wallis test, followed by Dunn’s multiple comparisons, p < 0.05.

SUPPLEMENTARY FIGURE S3 Quantitative analysis of 5HT4R mRNA expression in DSI and the colon of SCA7 Tg mice and controls revealed lack of desensitization of the receptor after 5 weeks of velusetrag treatment. Total RNA was isolated from DSI and the colon of all four groups of mice and used for cDNA synthesis. RT-qPCR reactions with specific primers for 5HT4R and GAPDH as internal control were performed as described in the Methods section. Values in the graph show relative mRNA expression for 5HT4R and are presented as the mean ± SEM, n = 10, one-way non-parametric ANOVA, Kruskal–Wallis test, followed by Dunn’s multiple comparisons, p < 0.05.

## Abbreviations

ATXN7, ataxin-7; CIPO, chronic intestinal pseudo-obstruction; DSI, distal small intestine; NIID, neuronal intranuclear inclusion disease; ENS, enteric nervous system; MNGIE, mitochondrial neurogastrointestinal encephalopathy syndrome; SCA7, spinocerebellar ataxia type 7; NIIs, neuronal intranuclear inclusions; IPAN, intrinsic primary afferent neuron; IACUC, Institutional Animal Care And Use Committee; SEM, standard error of the mean; °C, degree centigrade; SPF, specific pathogen free; PO, per os; Ct, cycle threshold.
