# Supplementary figures and images for: The 5HT4R agonist velusetrag efficacy on neuropathic chronic intestinal pseudo-obstruction in PrP-SCA7-92Q transgenic mice
Source: Front Pharmacol. 2024 Jul 30;15:1411642. doi: 10.3389/fphar.2024.1411642 (PMC11319301; doi:10.3389/fphar.2024.1411642)

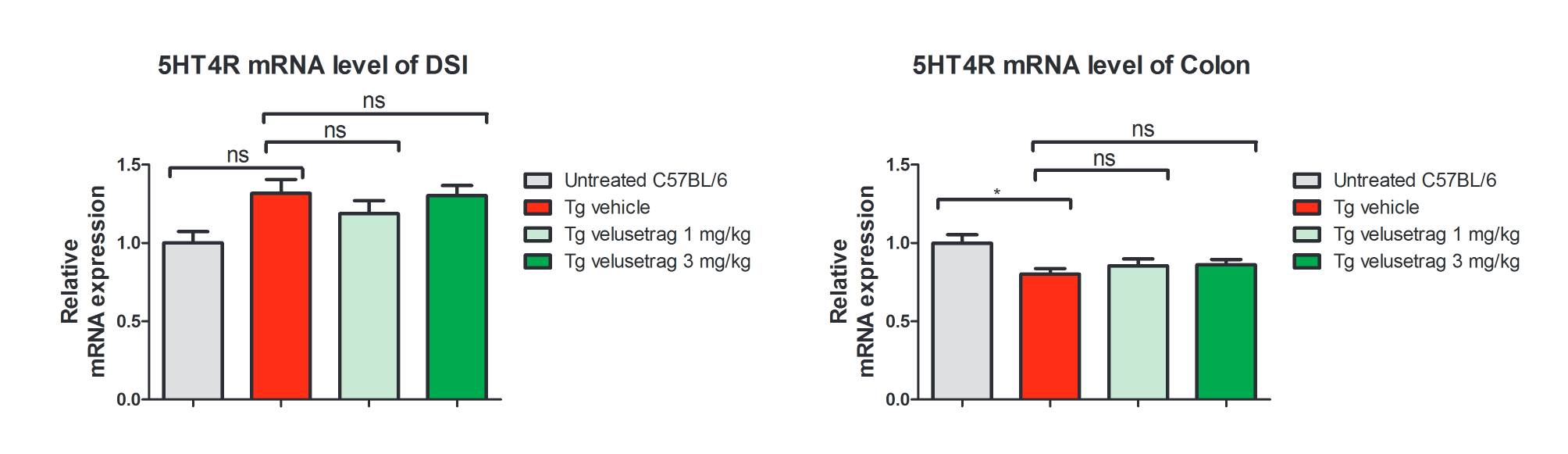

Supplement: Supplementary file 1 [file Image3.JPEG]

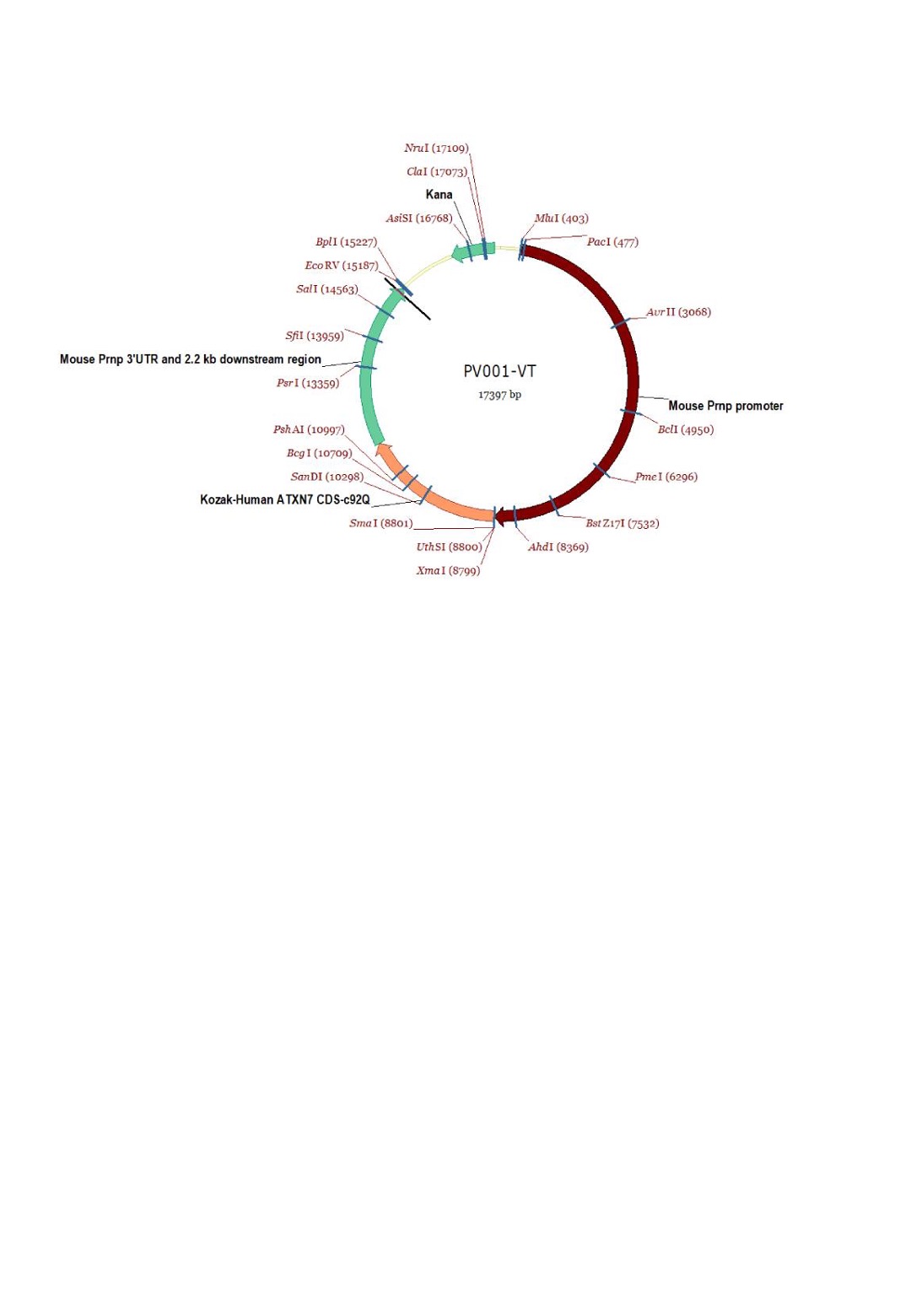

Supplement: Supplementary file 2 [file Image1.JPEG]

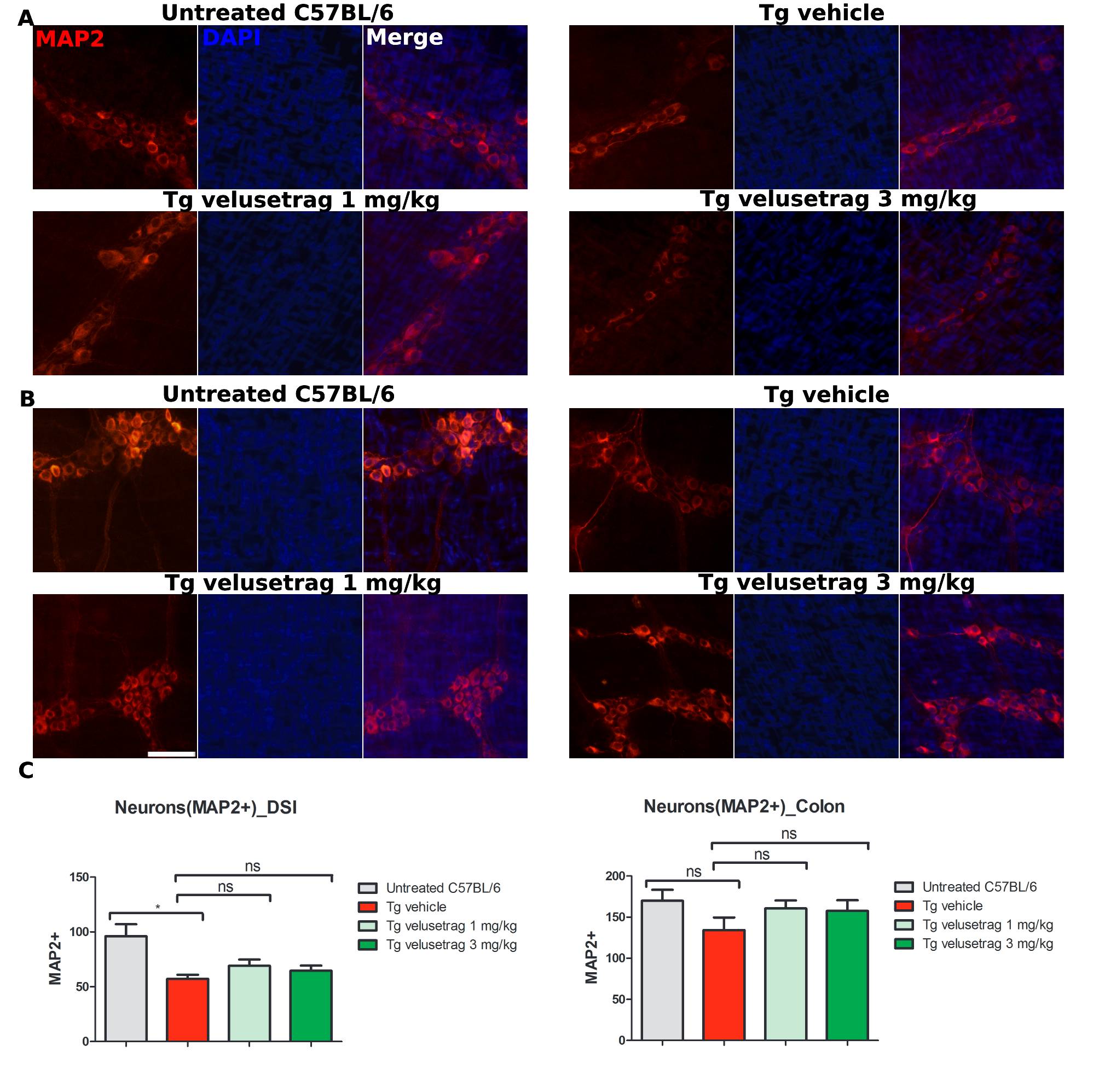

Supplement: Supplementary file 3 [file Image2.JPEG]
